# Intrahepatic Sarcomatous Cholangiocarcinoma: Case Report and Review of the Literature

**DOI:** 10.1155/2018/3862575

**Published:** 2018-01-09

**Authors:** Thana Boonsinsukh, Vichit Viriyaroj, Thammanij Rookkachart, Therdkiat Trongwongsa

**Affiliations:** ^1^Department of Surgery, Faculty of Medicine, Srinakharinwirot University, Ongkharak, Nakhon Nayok, Thailand; ^2^Department of Pathology, Faculty of Medicine, Srinakharinwirot University, Ongkharak, Nakhon Nayok, Thailand

## Abstract

The authors report a case of a patient with intrahepatic sarcomatous cholangiocarcinoma. A 45-year-old Thai man presented with a 3-month history of right upper abdominal pain. CT scan revealed hepatomegaly with a 6.5 cm hypovascular soft tissue density mass in the right lobe and showed mild delayed enhancement. On exploratory laparotomy, the tumor adherent to right diaphragm was found. We performed right hepatectomy, partial resection of right diaphragm, and cholecystectomy. The immunohistological results suggested “sarcomatous intrahepatic cholangiocarcinoma.” The tumor was recurrent in 5 months after operation and unresectable. Therefore, the treatment in this patient was supportive care. He died 11 months after his initial presentation. The literature reviews showed that intrahepatic sarcomatous cholangiocarcinoma is aggressive malignant with poor prognosis. Early detection, radical resection, and careful follow-up would be the treatment for the favorable prognosis.

## 1. Introduction

Sarcomatoid features are occasionally seen in various types of epithelial tumors such as renal cell carcinoma, squamous cell carcinoma, and adenocarcinoma of the lung [1]. Most sarcomatoid carcinomas in liver are sarcomatoid hepatocellular carcinoma [2, 3]. Although sarcomatoid cholangiocarcinoma is a rare entity, once found will often lead to poor prognosis. The clinical, imaging, and pathologic information regarding this tumor is still limited. It was difficult to establish the definite diagnosis in preoperation without the help of pathological reports. The optimal treatment of this rare lesion is unclear. We present here a case of intrahepatic sarcomatous cholangiocarcinoma, which was confirmed based on pathology.

## 2. Case Report

**Figure 1:**
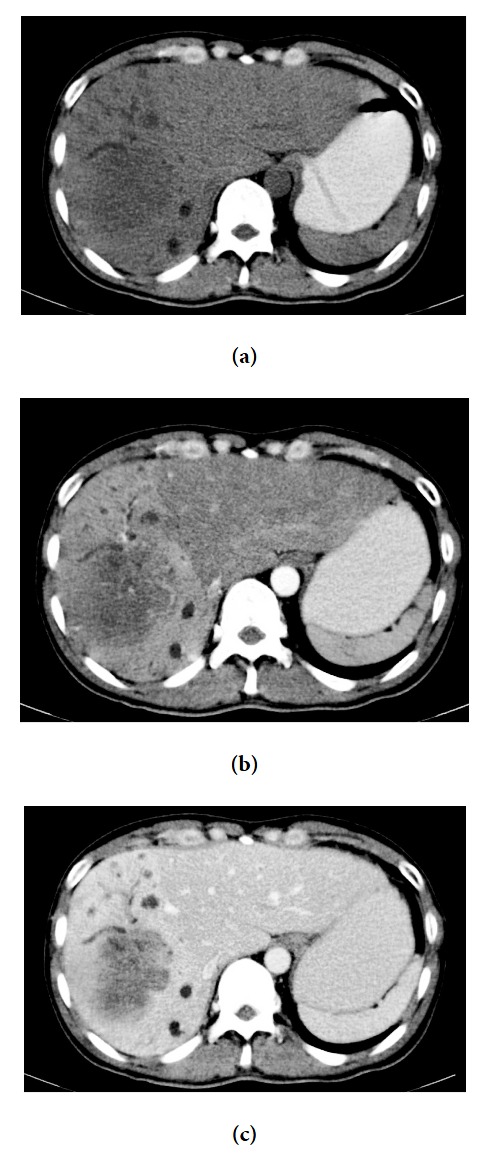
Computed tomographic scan showed tumor (a) hypoattenuating in precontrast phase, (b) no enhancement in arterial phase, and (c) mild enhancement in portal venous phase.

A 45-year-old Thai man presented with a 3-month history of right upper abdominal pain, weakness, loss of appetite, weight loss, and afebrile. He had no underlying disease. Ultrasonography showed ill-defined heterogeneous hypoechoic mass size 4.6 × 7.0 cm in the right lobe liver with dilated peripheral duct at the right lobe. Computed tomography (CT) scan revealed hepatomegaly with a 6.5 cm hypovascular soft tissue density mass in the right lobe and showed mild delayed enhancement (Figure 1). Multiple gallstones were seen in gallbladder, and lymph nodes were not enlarged.

Laboratory findings were as follows: total bilirubin, 3.31 mg/dL (normal range 0.3–1.2); serum alanine aminotransferase, 113 U/L (normal range 0–40); serum aspartate transaminase, 114 U/L (normal range 0–40); serum alkaline phosphates, 145 U/L (normal range 40–129); serum carcinoembryonic antigen (CEA), 2.8 ng/mL (normal range < 5.0 mg/mL); alpha-fetoprotein (AFP), 0.7 ng/mL (normal range < 9.6 ng·mL); and serum carbohydrate antigen 19-9 (CA 19-9), 42.2 U/mL (normal range < 39 U/mL). Serum markers for hepatitis C was positive and negative for hepatitis B.

**Figure 2:**
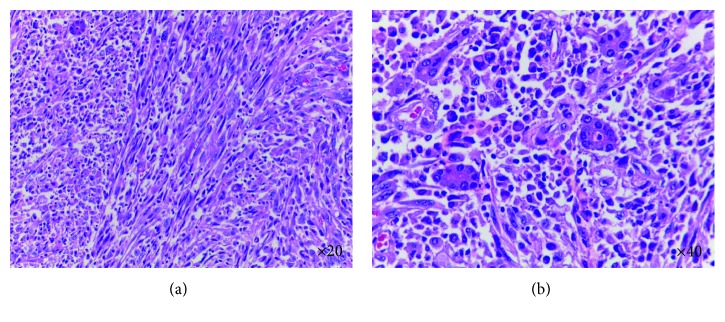
Histological appearance of the tumor showed malignant spindle cell (H&E staining).

On exploratory laparotomy, the tumor adherent to right diaphragm was found. We performed right hepatectomy, partial resection of right diaphragm, and cholecystectomy. The resected hepatic showed a 9 × 6 × 5 cm light brown tumor and adhered to diaphragm. Light microscopic examination of the tumor revealed an infiltrative tumor. The tumor mainly consists of spindle cell arranged in fascicles and storiform patterns; they had few scattered glandular formation (Figure 2). The tumor cells had moderately pleomorphic and hyperchromatic nuclei. Mitoses were 8/10 HPFs. Necrosis was noted. The tumor was free of resection margin, no lymphovascular invasion, no capsular invasion, and no diaphragm involvement. The remaining liver tissue showed noncirrhotic liver, and gallbladder showed chronic inflammatory cells infiltrate in the wall.

Immunohistochemical examinations demonstrated that the carcinoma cell was positive for vimentin, AE1/AE3 (focal), CAM5.2 (focal), CK7 (focal), and CK19 (focal) but negative for EMA, S-100, SMA, desmin, H-caldesmon, DOG-1, HepPar-1, glypican-3, CD34, CD117, PDGFR-Alpha, CK20, bile stain, iron, and mucicarmine. These immunohistological results suggested “sarcomatous intrahepatic cholangiocarcinoma.” The tumor was recurrent in 5 months after operation and unresectable. Therefore, the treatment in this patient was supportive care. He died 11 months after his initial presentation.

## 3. Discussion

Sarcomatous change in hepatocellular has been reported in about 3.9–9.4% of autopsy cases [2, 3]. The sarcomatous cholangiocarcinoma is extremely rare. Baek et al. [4] found only 11 cases of this tumor in the past 17 years. Therefore, clinical data on the prognosis and treatment are very limited, and the pathogenesis is unclear. Matsuo et al. [5] reported that sarcomatous cholangiocarcinoma should be distinguished from the coexistence of cholangiocarcinoma and hepatic sarcoma, as the main part of tumor consisted of pleomorphic spindle and cholangiocarcinoma intermingled in part. Boonsakan et al. [6] suggested that the exposure to the chemical carcinogen nitrosamine leads to sarcomatous transformation of the carcinoma. Kim et al. [7] reported that hepatolithiasis is considered as a cause of sarcomatous cholangiocarcinoma. Repeated episodes of cholangitis due to hepatolithiasis are considered to induce proliferative epithelial changes and accelerate tumorigenesis.

Literature reviews [4–18] showed most cases of sarcomatous cholangiocarcinoma that have been Asian. The average age of patients was 65 years old, ranging from 37 to 84 years. There have been equal male-to-female ratio and they did not show relationship between tumor and hepatitis virus infection (HBV and HCV) or cirrhosis. Most symptoms were presented with abdominal pain. Other symptoms were fatigue, fever, jaundice, weight loss, and accidental finding from ultrasound or CT. Intrahepatic sarcomatous cholangiocarcinoma generally presents extreme hypovascularity or low attenuated lesion with peripheral enhancement in delayed phase same as liver abscess on CT [4, 8]. Previous literature reported CA 19-9 level was within normal range, which is similar to our case, where CA 19-9 level was slightly increased (42.2 U/mL). However, there were some reports that showed CA 19-9 level was increased in sarcomatous cholangiocarcinoma [4, 7, 9]. No reports showed that CEA and AFP levels were sensitive for this tumor. Tumors ranged in diameter from 3.4 to 22 cm (mean 8.3 cm).

Radical resection was a strong option for treatment of this tumor. The survival rate of patients with surgical resection was significantly higher than that in patients without surgical resection [9]. Transarterial chemoembolization (TACE) may be beneficial for sarcomatous hepatocellular but not in the sarcomatous cholangiocarcinoma, where the survival rate was not improved [10]. There is no information available about the optimal adjunctive treatment after initial surgical resection. The effectiveness of chemotherapy and radiation therapy has not been extensively investigated. However, Malhotra et al. [11] demonstrated that combined chemotherapy with gemcitabine and cisplatin is the potential treatment option in sarcomatous cholangiocarcinoma after surgical resection, and Lin and Huang [12] reported that chemotherapy with doxorubicin and ifosfamide can be a viable therapeutic option for this recurrent tumor. As compared to intrahepatic cholangiocarcinoma, the prognosis for intrahepatic sarcomatoid cholangiocarcinoma is worse [19, 20], as there was aggressive spreading, widespread metastasis, and high recurrent rate. Most survival time with surgical resection was 3–6 months, but some reports showed more than 3 years [10, 11, 13].

In conclusion, intrahepatic sarcomatous cholangiocarcinoma is aggressive malignant with poor prognosis. Early detection, radical resection, and careful follow-up would be the treatment for the favorable prognosis. Adjuvant chemotherapy and radiation therapy are unclear. More epidemiological and pathological data will be further required to determine the appropriate surgical indication for this tumor.
